# An Introduction to Magnetic Resonance Imaging

**Published:** 1988-05

**Authors:** Terry Beddoe, Paul Goddard

**Affiliations:** Chairman of the Bristol MRI Scanner Fund; Trustee of the Bristol MRI Scanner Fund


Established 1883

BRISTOL MEDICO-CHIRURGICAL JOURNAL

Clinical Press

### An Introduction to Magnetic Resonance Imaging

**Terry Beddoe**
Chairman of the Bristol MRI Scanner Fund

**Paul Goddard**
Trustee of the Bristol MRI Scanner Fund

In 1976 and 1977 the first results from Magnetic Resonance Imaging appeared in the press. The images, of a finger and of an orange, were from an experimental system in Nottingham and were published in the British Journal of Radiology. Following this, original seminal work was also carried out at the Hammersmith Hospital and in Aberdeen.

A major revolution in Diagnostic Imaging was underway!

Many hundreds of scanners have now been built and installed in hospitals all round the world and the clinical results have been remarkable.

In 1984 a committee was set up by Radiologists from the three Bristol District Hospitals to look into the possibility of providing Magnetic Resonance Imaging for Bristol. In October 1985 Mr John James, after liaision with Dr Terry Beddoe and the radiologists from the original commitee, very generously gave the three District Hospitals the gift of a Picker 0.5 T superconductive MRI scanner. For this gift and for their subsequent support, the trustees of the Bristol MRI Scanner Fund would like to record their appreciation to Mr John James, Miss Pennington and the Trustees of the John James Trust.

Since that time the people of Bristol and the South West have helped the Bristol MRI Scanner Fund to raise the astonishing sum of £700,000. The groups donating to the fund range from pubs to churches and include Rotary, Freemasons, Round Table and Lions Clubs, Schools and Young Farmers clubs. The Salvation Army, various cricket clubs and the White Lion public house all raised many thousands of pounds and deserve special mention. Various hospital charities have donated large sums for which we are very grateful and individuals who should be mentioned include Mrs Miles, Mr Hugh Coakham and Dr Frank Ross.

We have had many generous donations from families in memory of relatives who have died and thousands of individual donations ranging from 10p to thousands of pounds.

We have only mentioned a small number of the fund raisers and organisations who have supported us so generously—there are many of them and their ingenuity and enthusiasm is amazing—we can never really express our grateful thanks to so many people.

The fund raising is not, however, over. We still have to raise around £300,000 in order to fund the machine—or perhaps even more since the "phasing in" agreement with the Region and the three local District Authorities does appear to be in some jeopardy.

In January 1988 a meeting of the Bristol Medico-Chirurgical Society was dedicated to Magnetic Resonance Imaging from the Bristol MRI Scanner. The meeting was a great success and the idea thus arose of a special issue of the journal dedicated to MRI.

This issue is a joyful testament to the British originators of MRI and to the people of Bristol and the South West who have so generously helped us.

It is also a record of the first 1000 patients to be scanned on the Bristol MRI Scanner.

**Acknowledgements for the Special Issue.**
The trustees of the Bristol MRI Scanner Fund would like to thank the sponsors of this special edition of the Medico-Chirurgical Journal:
Picker International Ltd
Schering Health Care Products
Dupont

